# Assessing Volumetric Absorptive Microsampling Coupled with Stable Isotope Dilution Assay and Liquid Chromatography–Tandem Mass Spectrometry as Potential Diagnostic Tool for Whole Blood 5-Methyltetrahydrofolic Acid

**DOI:** 10.3389/fnut.2017.00009

**Published:** 2017-04-18

**Authors:** Markus Kopp, Michael Rychlik

**Affiliations:** ^1^Chair of Analytical Food Chemistry, Technische Universität München, Freising, Germany; ^2^Institute for Food & Health, Technical University of Munich, Freising, Germany; ^3^Centre for Nutrition and Food Sciences, Queensland Alliance for Agriculture and Food Innovation (QAAFI), University of Queensland, Brisbane, Australia

**Keywords:** folate status, 5-methyltetrahydrofolic acid, volumetric absorptive microsampler, Mitra™, dried blood

## Abstract

Volumetric absorptive microsamplers (VAMS) have been developed recently as a promising tool for clinical blood sampling. Compared to dried blood spot samples analyzed by accurate stable isotope dilution assays (SIDAs), the new technique could provide further substantial miniaturizing of folate assays by eliminating hematocrit effects and uneven analyte distribution within the sample. Herein, we present a miniaturized SIDA coupled with LC-MS/MS measurement of 5-methyltetrahydrofolic acid as main folate vitamer in whole blood (WB) using [^13^C_5_]-5-methyltetrahydrofolic acid as internal standard. Elution and extraction of only 10.8 μL-dried WB were carried out by centrifugation followed by enzymatic treatment for polyglutamate deconjugation. Matrix separation was achieved by heating and centrifugation. To verify applicability, WB folate status of 11 volunteers was screened. Limits of detection and limits of quantitation were 9 and 26 nmol·L^−1^, respectively, which is sufficiently low for screening folate status. Recoveries were 97 (±5.8), 99 (±2.8), and 96 (±6.1)% for 800, 400, and 200 nmol L^−1^ 5-methyltetrahydrofolic acid, respectively. Precision of the LC-MS/MS instrument and inter-assay precision trials revealed CVs of 8.1 and 3.5% (294 nmol L^−1^), respectively, thus confirming reproducible and precise quantitation. Compared to fresh WB, no significant degradation of 5-methyltetrahydrofolate was observed after 2.5 h of drying at room temperature. VAMS 5-CH_3_-H_4_folate was stable for at least 3 weeks at −20°C. In our pilot study, accurate and diagnostically conclusive determination of folate status was verified. Nevertheless, blood sampling should be performed by trained individuals to avoid substantial errors concerning the absorbed volume. Endogenous folate in rat serum and chicken pancreas caused a significant background especially at low blood 5-CH_3_-H_4_folate levels and, thus, for polyglutamate deconjugation, these background folates or alternative mixtures need to be removed. The superior feasibility of a minimized blood collection with VAMS allows further progress regarding time- and cost-effective methodologies in newborn or population screenings for 5-methyltetrahydrofolate status. Further steps toward minimization could include an automated assay coupled with UPLC-MS/MS.

## Introduction

Folates are essential micronutrients, which are directly involved in purine and pyrimidine synthesis (1) and vitamin B_12_ mediated remethylation of homocysteine to methionine. The latter ensures maintenance of one-carbon metabolism and methylation of essential molecules like DNA, proteins, phospholipids, or neurotransmitters (2). Folate deficiency is a potential risk factor for inborn errors like spina bifida and anencephaly (3, 4) or diseases like Alzheimer’s (5, 6) and cancer (7, 8). Folate deficiency can also be due to C677T single nucleotide polymorphism in the methylenetetrahydrofolate reductase polymorphism gene, which encodes a thermally less stable enzyme with reduced activity (9). As a result, reduced transformation of 5,10-methylenetetrahydrofolic acid to 5-methyltetrahydrofolate (5-CH_3_-H_4_folate) leads to higher levels of circulating non-methylated folates such as 5,10-methylenetetrahydrofolic acid, 5-formyltetrahydrofolic acid, and tetrahydrofolic acid in erythrocytes (10). Erythrocyte folate, in particular, 5-CH_3_-H_4_folate (11), is a reliable marker for the determination of folate status (12) and its deficiency (13) in the body stores. Red blood cells are not influenced by recent dietary intake and maintain its folate content over 4-month lifespan (14). 5-CH_3_-H_4_folate represents the main vitamer in erythrocytes with reduced concentrations in humans with folate deficiency or increased levels of non-5-CH_3_-H_4_folate related to MTHFR C677T polymorphism (10).

Sensitive determination of folate status in erythrocytes or whole blood (WB) *via* LC-MS/MS requires several time-consuming purification steps, which include solid-phase extraction (SPE) (11, 15). However, little effort has been made to minimize WB assays (16–18) because plasma folate has been preferred as the marker of choice to target folate status and deficiency instead of erythrocyte folate, which reflects folate status in the same manner (13, 19). Two principal drawbacks were the complex matrix of erythrocytes, which requires more extraction steps and a longer time, resulting in higher costs (13).

Nevertheless, simplified blood collection technologies with low blood volume (<100 μL) and fast sample processing are in great demand (20). The development of a simple dried blood spot (DBS) method for the screening of phenylketonuria by Guthrie and Susi (21) led to the establishment of a fast-growing field, using dried blood and plasma sampling methods for drug monitoring and health assessment. Predictive diagnostic values can be obtained from DBS (16, 18) in combination with sensitive technologies, while costs for transport, storage (22), and surveillance staff can be minimized (23). Nevertheless, hematocrit bias within the spot (24–26) influencing spreadability of the applied blood volume due to increasing viscosity (27) has to be considered. Moreover, uneven analyte distribution caused by chromatographic effects as observed for vitamin D (28) and retinol (29) or dependence of punch volume on spotted blood volume (28) have to be carefully evaluated during methodology development.

Three methods describe the assessment of 5-CH_3_-H_4_folate or folate in 50 μL (16–18) or 100 μL (17) WB DBS. Ultrasonic treatment of DBS and folate analysis by microbiological assays were the first approaches (16, 17) to assess folate status followed by our stable isotope dilution assay (SIDA) coupled with sensitive LC-MS/MS measurement (18). For WB 5-CH_3_-H_4_folate determination in DBS, we used the entire 50 μL spot as we wanted to avoid imprecision due to hematocrit bias and analyte distribution. Unfortunately, this approach requires precise sampling performed by volunteers, as they must handle microlancets and capillary tubes to spot a defined volume (18). In contrast, conventional punching of DBS disks of a few square millimeter out of a large spot requires the determination of the homogeneity of red blood cells and the exact volume of the disk.

Recently, a Mitra™ volumetric absorptive microsamplers (VAMS) for research use only [Mitra (RUO) VAMS] has been developed to facilitate blood sampling and to overcome hematocrit effects along with reducing costs and ensuring reproducible blood collection with a defined blood volume (22). For the determination of WB 5-CH_3_-H_4_folate in VAMS, the 10 μL sampling device could provide further standardization and miniaturization of state-of-the-art methodology. A better sampling procedure and, in particular, time reduction with reduced sample volume might provide much more consistent and reproducible work flows. Therefore, we developed and validated a low sample volume SIDA coupled with LC-MS/MS measurement for WB 5-CH_3_-H_4_folate with the goal of optimizing cost-efficiency, sensitivity, and sample throughput.

## Materials and Methods

### Chemicals

All chemicals used have been published recently (18). Rat serum and chicken pancreas containing γ-glutamyl hydrolase (EC 3.4.19.9) were obtained from Biozol (Eching, Germany) and Difco (Sparks, MD, USA), respectively. Mitra (RUO) VAMS were purchased from Neoteryx (Torrance, CA, USA). PVDF membrane filters (13 mm, 0.22 μm) were obtained from Berrytec (Grünwald, Germany). [^13^C_5_]-5-CH_3_-H_4_folate was purchased from Merck (Schaffhausen, Switzerland). Disposable micro lancets (Accu Check Safe-T-Pro Plus) were obtained from Roche Diagnostics (Mannheim, Germany). Desiccant bags were obtained from Clariant (Muttenz, Switzerland). Strata SAX cartridges (quaternary amine, 100 mg, 1 mL) were obtained from Phenomenex (Aschaffenburg, Germany).

### Solutions

Extraction buffer for VAMS comprised 0.1% Triton X-100 in a 20 g L^−1^ ascorbic acid and 2-(*N*-morpholino)ethanesulfonic acid hydrate (MES) (200 mmol L^−1^) solution with 1 g L^−1^ DTT and was adjusted to pH 5 with 7.5 M NaOH. Phosphate buffer (100 mmol L^−1^) was prepared by adjusting a 100 mmol L^−1^ disodium hydrogen phosphate aqueous solution with a potassium dihydrogen phosphate (100 mmol L^−1^) aqueous solution to pH 7.0. The equilibration buffer for the SAX cartridges was prepared by adding 0.02 g DTT to diluted phosphate buffer (10 mmol L^−1^). Further, the eluting solution was a mixture of aqueous sodium chloride (5%) and aqueous sodium acetate (100 mmol L^−1^) containing 0.1 g DTT and ascorbic acid (1%). A chicken pancreas suspension for folylpolyglutamate deconjugation was prepared by stirring chicken pancreas (10 mg) in aqueous phosphate buffer solution (60 mL, 100 mmol L^−1^) containing 1% ascorbic acid adjusted to pH 7 with 7.5 M NaOH. Rat serum was aliquoted and stored at −20°C without further dilution. [^13^C_5_]-5-CH_3_-H_4_folate solution (internal standard) was prepared by diluting 5 mg of [^13^C_5_]-5-CH_3_-H_4_folate to a final concentration of 57.5 mmol L^−1^ with extraction buffer. The standard solution was stored at −20°C.

### Sampling Procedure and Ethical Permission

**Figure 1:**
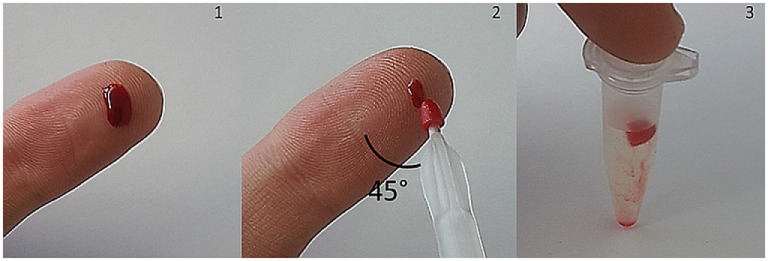
**Blood collection with volumetric absorptive microsamplers (VAMS) after index finger puncture**. Approximately 10 μL of whole blood are absorbed by the microsampling device. After drying at room temperature, VAMS can be stored at −20°C with desiccant or removed and suspended for analysis.

A typical sampling procedure is shown in Figure 1. Blood sampling was performed by index finger puncture using a disposable micro lancet. The first drop was discarded and the second drop appearing was used. “Milking” has to be avoided because tissue fluids may lead to a dilution of blood. Afterward, the finger was placed on a flat surface (e.g., table) [Figure 1 (1)]. Finger blood was collected by slightly immersing the VAMS into the blood drop from above (as recommended by the manufacturer). Between sampling device and finger/surface an angle of 45° is recommended [Figure 1 (2), viewed from above]. After removing the tip, the clamshell containing four devices was closed and dried in resealable bags with 1 g of desiccant for 2.5 h. Finally, VAMS were stored at −20°C until extraction. Before extraction, the tip was stripped off with tweezers into a 1.5 mL Eppendorf tube [Figure 1 (3)].

Eleven healthy, non-smoking Caucasian subjects participated in the pilot study. Ethical permission was obtained by the Ethics Committee of the Faculty of Medicine of the Technische Universität München (project 4031/11). The study was carried out in accordance with the recommendations of the Ethics Committee given above with written informed consent from all subjects. All subjects gave written informed consent in accordance with the Declaration of Helsinki.

### Sample Extraction

Prior to extraction, the tips were removed from the VAMS, placed in 1.5 mL Eppendorf tubes, and spiked with 100 μL of the [^13^C_5_]-5-CH_3_-H_4_folate solution (57.5 nmol L^−1^). Then, 400 μL of extraction buffer was added to the sample. The suspension was vortexed for 2 s and centrifuged for 10 min at 15,400 *g* to elute the WB from the tip swimming on top of the suspension. For polyglutamate deconjugation, 75 μL of rat serum and 500 μL of chicken pancreas suspension were added to the WB eluate. After further 5 s of vortexing, the samples were incubated for 60 min at 37°C in a vibrating water bath. Incubated samples were heated for 6 min at 100°C for protein precipitation. Samples were then cooled for 10 min in a freezer (−20°C) and centrifuged for further 10 min at 15,400 *g*. Supernatants were filtered with a PVDF syringe filter.

### Endogenous Plasma Conjugase Activity and SPE

Human plasma deconjugase activity was determined by extracting three VAMS according to the extraction procedure detailed above using 75 μL of rat serum and 500 μL of chicken pancreas suspension for deconjugation (1). Further three VAMS were extracted without additional enzymes (2). Incubated samples (1, 2) were heated for 6 min at 100°C for protein precipitation and subjected to SPE cleanup as follows. VAMS extracts were purified by SPE using a 12-port vacuum manifold (Merck) equipped with Strata SAX cartridges (quaternary amine, 100 mg, 1 mL). The cartridges were activated with two volumes of methanol and two volumes of equilibration buffer (pH 7), followed by the application of the respective VAMS extracts. Afterward, the cartridges were washed with two volumes of equilibration buffer and subsequently dried by vacuum suction. 5-CH_3_-H_4_folate was eluted with 0.5 mL eluting solution.

Additional three VAMS (3) were extracted analogously without enzymes and SPE.

### Determination of Endogenous Enzyme Folate

Endogenous 5-CH_3_-H_4_folate of the enzyme mixture was determined by extracting 75 μL of rat serum and 500 μL of chicken pancreas suspension in triplicate according to the extraction of samples. Endogenous 5-CH_3_-H_4_folate was subtracted from the samples after LC-MS/MS measurement.

### Validation of the Folate Assay

#### Liquid Chromatography–Tandem Mass Spectrometry

About 10 μL of the samples were measured using a Finnigan Surveyor Plus HPLC System coupled with a Triple Quadrupole TSQ Quantum Discovery Mass Spectrometer (Thermo Electron Corporation, Waltham, MA, USA) equipped with a Nucleosil 100-5 C18 EC 250/3 column (Macherey-Nagel, Düren, Germany). Aqueous formic acid (0.1%; eluent A) and acetonitrile containing 0.1% formic acid (eluent B) were used as eluents at a flow rate of 0.3 mL/min. Gradient elution started at 0% B, followed by a linear increase of B to 30% within 12 min and to 90% within a further 3 min. Subsequently, the mobile phase was held at 90% B for an additional 3 min before equilibrating the column after 2 min at 0% B for 13 min. Total run time including equilibration was 33 min. Multiple reaction monitoring (MRM) was carried out at the positive electrospray mode between 12 and 18 min. The MRM transitions were *m/z* 460 → 313/180 for 5-CH_3_-H_4_folate and *m/z* 465 → 313/180 for [^13^C_5_]-5-CH_3_-H_4_folate. MRM parameters are shown in Table 1.

**Table 1:** **Multiple reaction monitoring parameters for the quantitation of 5-CH_3_-H_4_folate**.

Analyte	Q1 (*m/z*)	Q3 (*m/z*)	CE	CID
5-CH_3_-H_4_folate	460	313	21	10
	460	180	38	10
[^13^C_5_]-5-CH_3_-H_4_folate	465	313	21	10
	465	180	38	10

*Source CID collision energy: 10; spray voltage 3,900 V; capillary temperature 320°C; capillary voltage 35 V; Q2 collision gas pressure*.

#### Determination of the Blood Volume Absorbed by VAMS

According to Spooner et al. (22), we assessed the exact volume absorbed by the VAMS using aliquots of 2 mL heparinized WB. After determining the initial weight of the blood sample, 10 μL of blood were removed with a calibrated pipet and the weight of the vial with the remaining blood was recorded. After five repetitions, the blood density was calculated using the following equation:

$$\text{Blood density}[\frac{\text{g}}{\text{mL}}]=\frac{\text{mean weight of blood in 10 μL aliquot}\;[\text{g}]}{10}\times 1,000.$$

The experiment was repeated with another, identical blood sample of known weight by immersing the VAMS tip until recommended loading was reached. After five repetitions, the absorbed blood volume was calculated as follows:

$$\text{volume absorbed by VAMS tip [mL]}=\text{mean weight of blood absorbed by tip [g] / blood density}\left[\frac{\text{g}}{\text{mL}}\right].$$

#### Calibration and Quantitation

The purity of unlabeled 5-CH_3_-H_4_folate used for obtaining an appropriate response was determined by measuring the analyte and folic acid as internal standard using high performance liquid chromatography coupled with diode array detection (HPLC-DAD) according to the method from a previous study (18). Calibrator solutions were prepared by mixing 100 μL of diluted unlabeled 5-CH_3_-H_4_folate stock solutions with 100 μL of a 57.5 nmol L^−1^ [^13^C_5_]-5-CH_3_-H_4_folate standard solution in molar ratios [*n*(*S*)/*n*(*A*)] of 6:1–0.6:1. About 875 μL of extraction buffer were added to the mixture to adjust for the final sample volume. Concentrations of the respective stock solutions are given in Table 2. Linear regression was used for the calibration function including the area ratios [*A*(*S*)/*A*(*A*)] obtained from LC-MS/MS measurement. The response equation was calculated as follows:

$$\frac{A\left(\text{labeled standard}\right)}{A\left(\text{analyte}\right)}=\text{Rf}\times \frac{n\left(\text{labeled standard}\right)}{n\left(\text{analyte}\right)}\;-\;b.$$

**Table 2:** **Concentration of calibrator solutions**.

Calibrator	c(5-CH_3_-H_4_folate) (nmol L^−1^)	n(5-CH_3_-H_4_folate) (nmol)	n(IS) (nmol)
1	100	0.0100	0.00575
2	90	0.0090	0.00575
3	80	0.0080	0.00575
4	70	0.0070	0.00575
5	60	0.0060	0.00575
6	50	0.0050	0.00575
7	45	0.0045	0.00575
8	40	0.0040	0.00575
9	35	0.0035	0.00575
10	30	0.0030	0.00575
11	25	0.0025	0.00575
12	20	0.0020	0.00575
13	15	0.0015	0.00575
14	10	0.0010	0.00575

*IS, internal standard [^13^C_5_]-5-CH_3_-H_4_folate*.

Consistency of the response was checked by measuring a randomly chosen *n*(*S*)/*n*(*A*) value in the linear range prior to sample analysis.

#### Theoretical Limits of Detection (LOD) and Limits of Quantitation (LOQ)

The LOD and LOQ in dried WB were determined in analogy to Hädrich and Vogelgesang (30). A WB surrogate was prepared in analogy to our recent studies from lyophilized egg white (cellular fraction) in a mixture of sunflower oil, aqueous sodium chloride, and lyophilized egg white (plasma fraction) (15, 18). Twelve VAMS were immersed into 10.0 μL of the surrogate until loading was reached. Samplers were dried for 2.5 h and then frozen at −20°C in bags with desiccant. For LOD and LOQ determination, the matrices were spiked with 5-CH_3_-H_4_folate at four different levels, starting at slightly above the estimated LOD and covering onefold to tenfold the amount of the analyte. After the addition of internal standard, the VAMS were extracted as aforementioned.

#### Matrix Effects

Two VAMS were loaded with fresh finger blood and extracted after addition of 100 μL [^13^C_5_]-5-CH_3_-H_4_folate. Furthermore, two blanks consisting of 10.0 μL distilled water were spiked with the internal standard and extracted analogously. Blood extracts and blanks were measured in alternate sequence. Matrix effects depicting the attenuation of the standard signal were calculated using the following equation:

$$\text{matrix effect}\;\left[\text{\%}\right]=\text{100\%}-\frac{\text{area IS in blood extract}}{\text{area IS in blank}}\times \text{100\%}.$$

#### Precision

Precision of the LC-MS/MS instrument (i.e., inter-injection variation) was determined by extracting one VAMS from freshly drawn EDTA-finger blood of three independent blood donors with 114, 306, and 409 nmol L^−1^ 5-CH_3_-H_4_folate, respectively, and performing three injections in a row.

For inter-assay (i.e., inter-day) precision, VAMS were prepared by immersing the tip in freshly drawn EDTA-finger blood (294 nmol L^−1^) of a male blood donor (28 years). Inter-assay precision was determined by analyzing VAMS in triplicate per day over 3 days within 3 weeks. Each sample was injected three times in a row, and the mean value of these three injections was used to eliminate inter-injection variation for each extraction. To further eliminate intra-day variation, the mean of the three extractions was calculated per day. The CV of inter-assay (i.e., inter-day) was then obtained from the means of day 1, 2, and 3.

#### Recoveries of SIDA

Nine VAMS were immersed into 10.0 μL of the blood surrogate and dried as aforementioned. Tips were suspended in triplicate in 10.0 μL of extraction buffer containing 8 (level I), 4 (level II), and 2 pmol (level III) 5-CH_3_-H_4_folate equivalent to blood concentrations of 800, 400, and 200 nmol L^−1^ 5-CH_3_-H_4_folate. After absorption of the spiked analyte, 100 μL of the [^13^C_5_]-labeled internal standard solution and 400 μL of extraction buffer were added to the samples. Extraction was performed as aforementioned. Recoveries were calculated as follows:

$$\text{recovery}\;\left[\text{\%}\right]=\frac{\text{recovered mean concentration of analyte}\;[\frac{\text{nmol}}{\text{L}}]}{\text{spiked concentration of analyte}\;[\frac{\text{nmol}}{\text{L}}]}\times \text{100\%}.$$

#### Stability of 5-CH_3_-H_4_folate during Air Drying at Room Temperature

To evaluate the necessity of an antioxidant for 5-CH_3_-H_4_folate preservation, we compared three aliquots of 10.0 μL freshly drawn EDTA-WB after forefinger puncture stored at 4°C for 2.5 h with three VAMS (10.8 μL) prepared from the same location and analyzed immediately after drying to evaluate the degradation of 5-CH_3_-H_4_folate by oxygen at room temperature during the 2.5 h drying process.

#### Stability of 5-CH_3_-H_4_folate in VAMS at −20°C

To assess the stability of VAMS stored at −20°C in resealable bags with desiccant over approximately 3 weeks, 12 VAMS were prepared and analyzed on day of preparation (day 0) and after 3, 14, and 17 days in triplicate.

#### Stability of 5-CH_3_-H_4_folate in Extracted Samples during LC-MS/MS Analysis

Analyte stability in autosampler vials at room temperature was determined by observation of the internal standard area during 64 injections. Injections 1 and 64 were a response mixture of 5-CH_3_-H_4_folate and the deuterated internal standard in extraction buffer. Samples 2–63 were blood extracts from blood donors. To prevent the extract from degradation by UV-light, we conducted our measurements under exclusion of light.

#### Comparison of VAMS and DBS Assay

An EDTA-blood sample of 200 μL freshly drawn blood from index finger puncture was divided in two aliquots. Aliquot 1 was used to prepare three 10.0 μL aliquots in Eppendorf tubes, which were stored in the refrigerator at 4°C. In addition, three VAMS (10.8 μL) were prepared and dried for 2.5 h. Three 20.0 μL aliquots of finger blood were prepared in Eppendorf tubes from aliquot 2 and stored in the refrigerator. Furthermore, three 20 μL DBS were prepared according to Kopp and Rychlik (18) with 20 μL capillaries. DBS and 20.0 μL WB were extracted according to Kopp and Rychlik (18). VAMS and 10.0 μL aliquots were subjected to the extraction procedure mentioned above. Results were compared for their consistency.

### Preliminary Applications in Human Studies—Determination of WB 5-CH_3_-H_4_folate Status

Female (*n* = 6) and male (*n* = 5) adults were examined for their folate status. The health status and lifestyle of the volunteers were not taken into account. Each subject delivered three VAMS.

### Data Analysis

All data analysis was performed using Xcalibur Software from Thermo Scientific (Waltham, MA, USA). Statistical analysis (Mandel-Test and two-sided *t*-test with *p* = 0.05) was performed using Excel 2013.

### Safety Precautions

Blood has to be considered as potentially infected with harmful pathogens, which can be transferred from person to person. Therefore, direct contact with these fluids must be strictly avoided.

## Results

Mitra™ (RUO) VAMS offer a feasible sampling method to obtain approximately 10 μL WB sample right after index finger puncture. The tip can be stripped off easily whereas whole DBS have to be punched out for folate analysis in a more time-consuming procedure. Analysis of 5-CH_3_-H_4_folate in DBS by means of SIDAs conducted as LC-MS/MS measurements proved to be the method of choice for accurate and precise evaluation of folate status in WB, as they compensate for matrix interferences and poor analyte stability (18). The method presented herein combines the benefits of both technologies to develop a first cost-effective, minimized, and fast sampling method to assess WB 5-CH_3_-H_4_folate forming the basis for future folate assays.

### Endogenous Plasma Conjugase Activity and SPE

**Figure 2:**
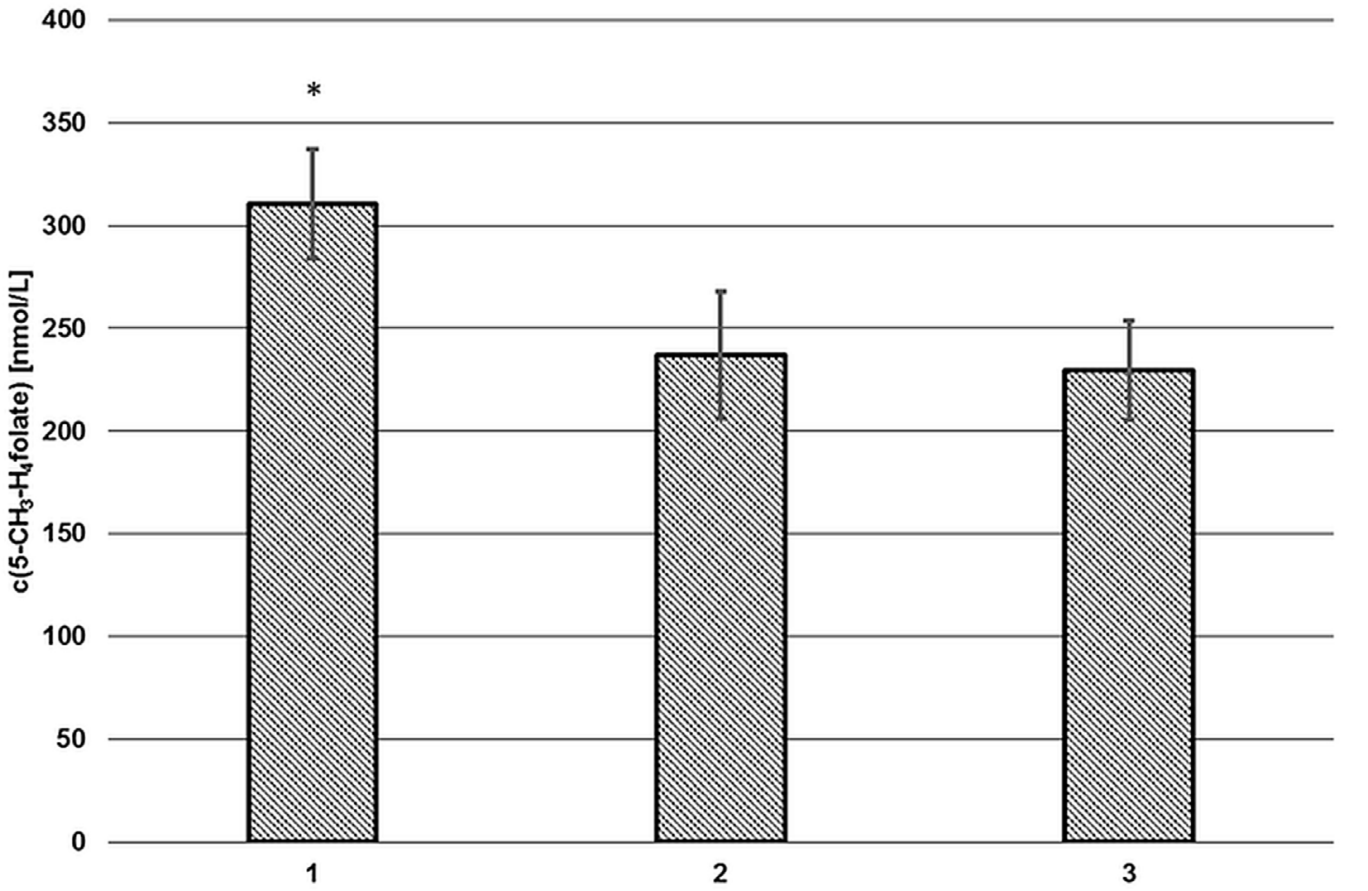
**Extraction of volumetric absorptive microsamplers with (1) and without additional enzymes (2), without solid phase extraction/enzymes (3)**. Mean ± SD (*n* = 3). *Significant difference (*p* = 0.05).

As depicted in Figure 2 (2, 3), endogenous human plasma deconjugase activity did not yield quantitative pteroylpolyglutamate deconjugation and significant traces of 5-CH_3_-H_4_PteGlu_2_ were still detectable after 60 min at 37°C. With additional exogenous deconjugase (1) significantly higher levels of 311 ± 27 nmol L^−1^ 5-CH_3_-H_4_folate were obtained compared to VAMS extracted without enzymes (237 ± 31 nmol L^−1^, −24%) (2), and without enzymes or SPE (229 ± 24 nmol L^−1^, −27%) (3) (*p* = 0.05). Therefore, the addition of exogenous enzymes from rat serum and chicken pancreas is urgently needed.

Volumetric absorptive microsamplers extracted with SPE (2) or without additional SPE cleanup (3) showed no significant difference (*p* = 0.05). Thus, the SPE step was omitted. Matrix effects were assessed separately (see “Matrix Effects”).

### Determination of Endogenous Enzyme Folate

**Figure 3:**
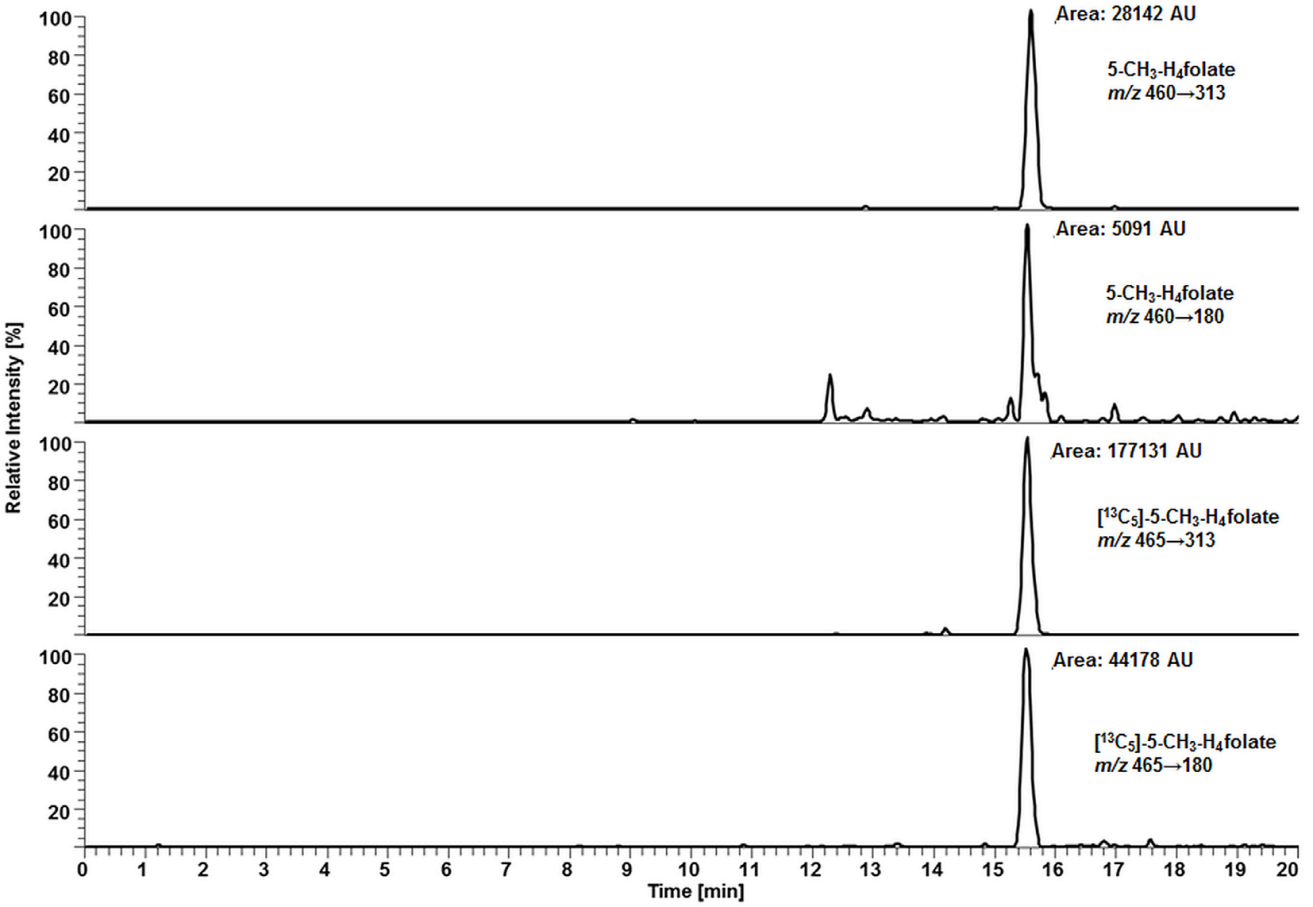
**Liquid chromatography–tandem mass spectrometry chromatogram of the enzyme mixture (75 μL rat serum and 500 μL chicken pancreas suspension)**.

**Figure 4:**
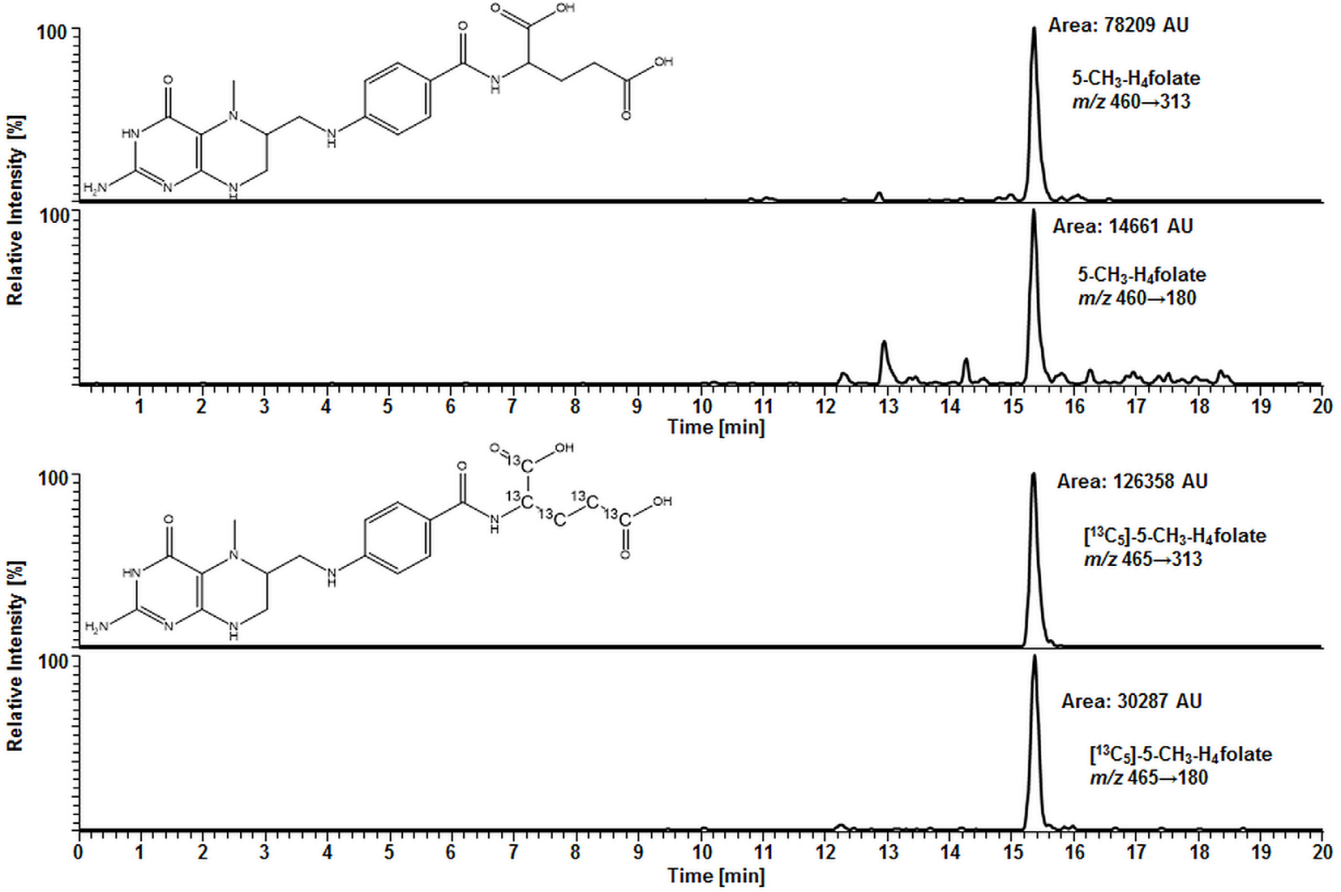
**Liquid chromatography–tandem mass spectrometry chromatogram in the multiple-reaction monitoring mode for volumetric absorptive microsamplers [10.8 μL whole blood sample (329 nmol L^−1^ 5-CH_3_-H_4_folate)]**.

For deconjugation of native polyglutamylated 5-CH_3_-H_4_folate, we used an enzyme mixture including rat serum and chicken pancreas suspension. As the mixture contains endogenous folate, we extracted the enzyme blank to check for 5-CH_3_-H_4_folate. The enzyme blank consisting of 75 μL rat serum and 500 μL chicken pancreas suspension contained 0.001 nmol 5-CH_3_-H_4_folate, which has to be subtracted after determination of WB 5-CH_3_-H_4_folate. A chromatogram of the extracted enzyme mixture with 1 pmol endogenous 5-CH_3_-H_4_folate is shown in Figure 3. The chromatogram of one VAMS of a male blood donor with 3.29 pmol total 5-CH_3_-H_4_folate (blood 5-CH_3_-H_4_folate and enzyme 5-CH_3_-H_4_folate) is shown in Figure 4. In this case, endogenous enzyme 5-CH_3_-H_4_folate contributes to approximately one-fourth of total 5-CH_3_-H_4_folate in the deconjugated sample.

### Method Validation

#### Determination of the Blood Volume Absorbed by VAMS

Based on the values in Table 3, a mean blood volume of 10.8 (±0.5) μL was determined to be absorbed by the VAMS sampler. Therefore, we confirmed the findings of Spooner et al. (22) who determined an average blood volume of 10.6 μL matching the certified value for the VAMS lot (10.5 μL). Based on these findings, 10.8 μL WB were used for the calculation of 5-CH_3_-H_4_folate from VAMS.

**Table 3:** **Calculation of the blood volume absorbed by volumetric absorptive microsamplers (VAMS)**.

Repetition	Weight of blood in 10 μL aliquot (g) (pipet)	Weight of blood absorbed by VAMS (g)
1	0.0109	0.0114
2	0.0096	0.0116
3	0.0122	0.0125
4	0.0110	0.0118
5	0.0105	0.0113
Mean weight (g)	0.0108	0.0117
Blood density (g/mL)	1.08	–
Volume absorbed	10.0	10.8

#### Calibration and Quantitation

The calibration function for 5-CH_3_-H_4_folate proved to be linear (Mandel-Test) covering molar ratios from 6:1 (representing 100 nmol L^−1^ 5-CH_3_-H_4_folate in WB) to 0.6:1 (representing 1,000 nmol L^−1^ 5-CH_3_-H_4_folate in WB) (*y* = 1.2739*x* − 0.0387, *R*^2^ = 0.9982).

#### Theoretical LOD and LOQ

Due to the ubiquitous occurrence of folate, we used a WB surrogate (15, 18) for the determination of LOD/LOQ and recoveries.

In our method described recently (18) using 50 μL DBS, we obtained a LOD and LOQ of 9.1 and 27 nmol L^−1^, respectively. Mönch and coworkers (15) determined a LOD of 12 nmol L^−1^ for 5-CH_3_-H_4_folate in 40 mg lyophilized erythrocytes. Using the spiking procedure proposed by Hädrich and Vogelgesang (30), LOD and LOQ for 10.8 μL VAMS were 9 and 26 nmol L^−1^ revealing similar sensitivity even after a 80% reduction of sample volume compared to the methods mentioned above. It has to be mentioned that LOD and LOQ were calculated on a theoretical basis because they were determined in absence of rat serum and chicken pancreas, which would cause a significant analyte background. When eluted from the tip, dried WB forms a transparent solution in extraction buffer, whereas DBS WB partly precipitates after centrifugation or ultrasonication. A typical MRM chromatogram is shown in Figure 4.

After 600 injections, retention time and peak shape remained stable. Therefore, we recommend the use of this column especially for labs in search for low-priced and robust accessories.

#### Matrix Effects

**Figure 5:**
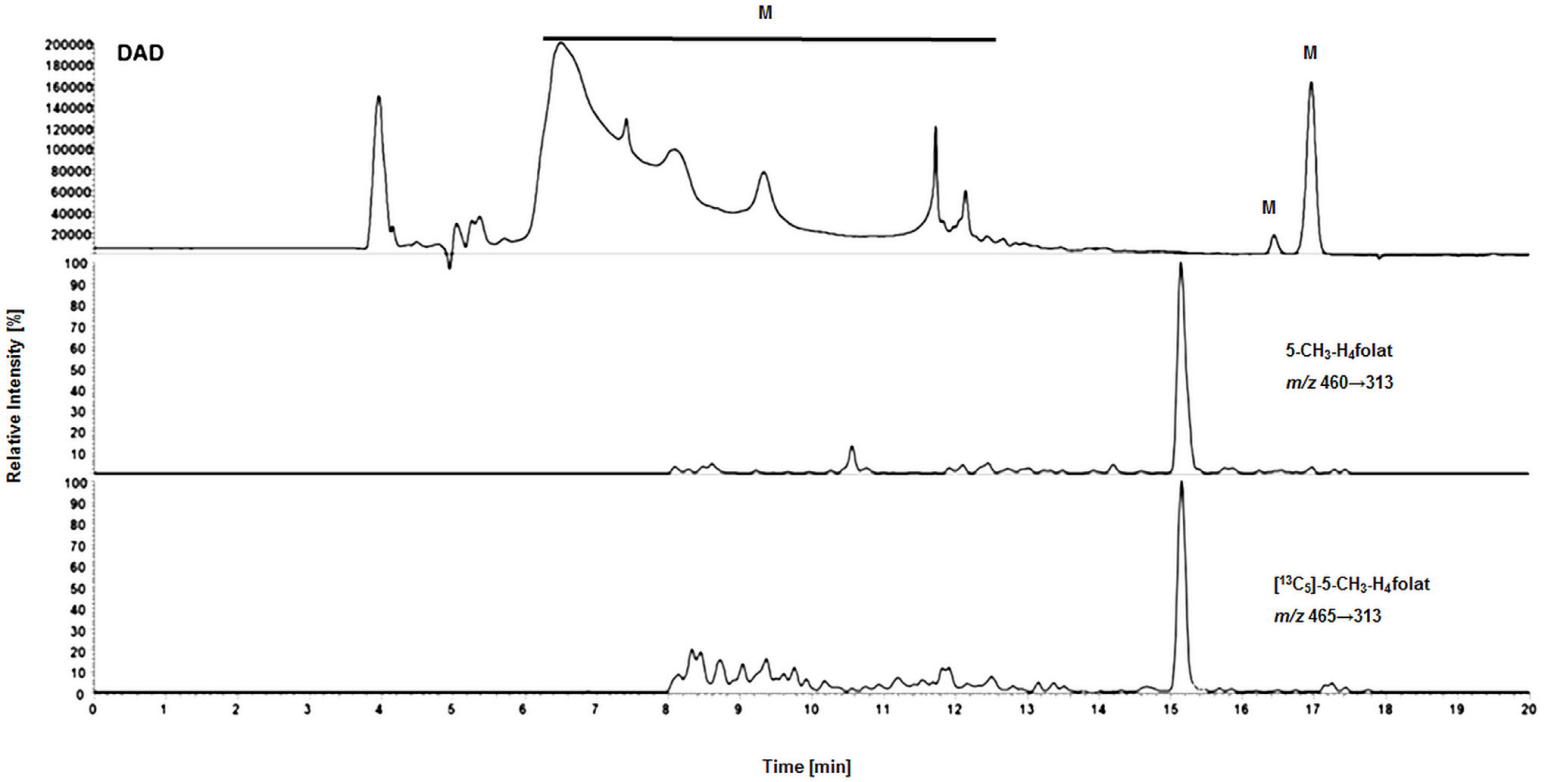
**Matrix effects during LC-MS/MS measurement of extracted volumetric absorptive microsamplers**. DAD (diode array detector), M (interfering compounds and inorganic salts from whole blood).

**Figure 6:**
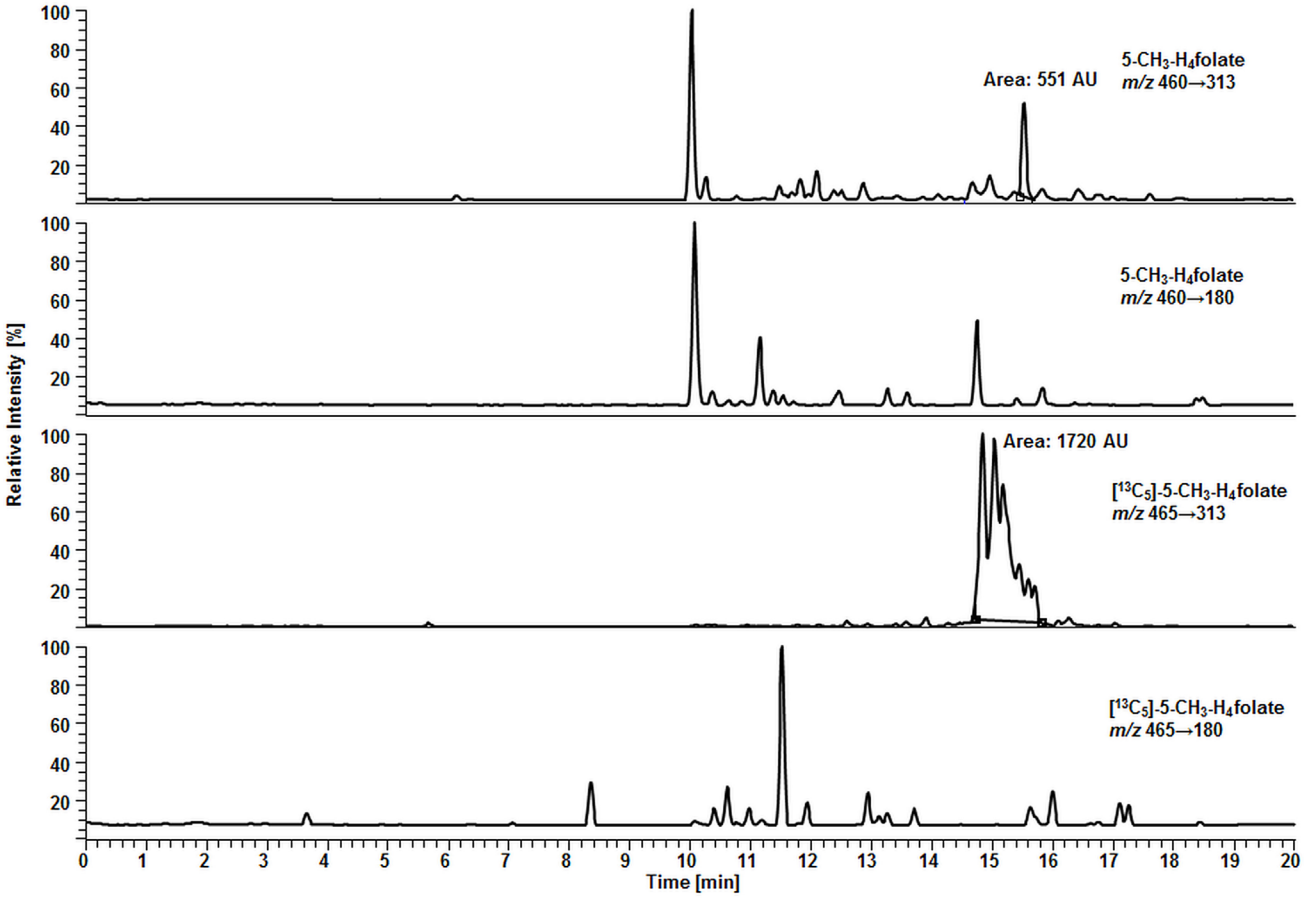
**Liquid chromatography–tandem mass spectrometry chromatogram of a water blank**.

**Figure 7:**
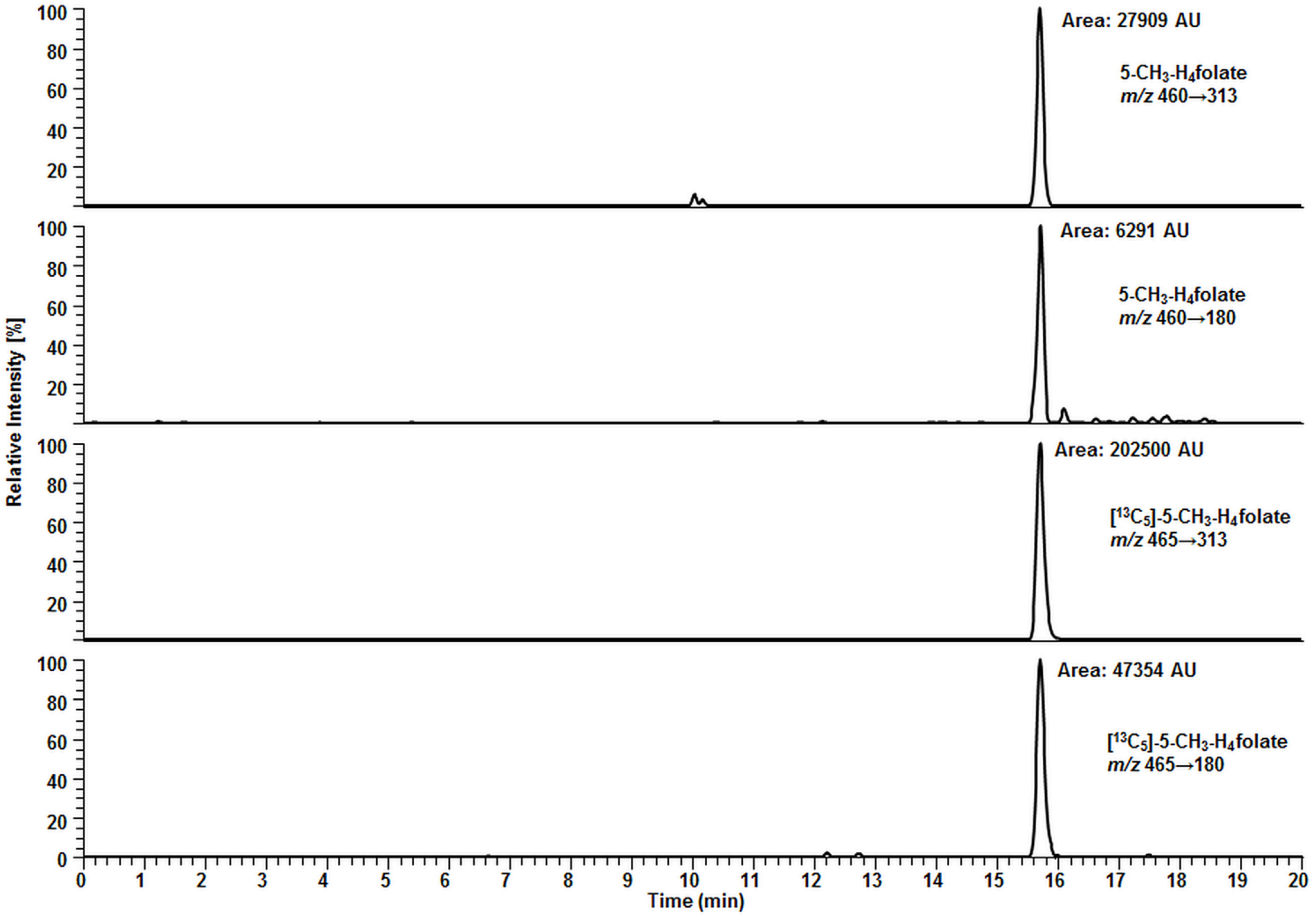
**Liquid chromatography–tandem mass spectrometry chromatogram of calibrator 14 (Table 2)**.

Surprisingly, we observed no matrix interferences with the analyte or standard signal after replacing the SPE by a simple centrifugation step. Areas of [^13^C_5_]-5-CH_3_-H_4_folate were approx. 22,000 and 24,000 AU for blood extract 1 and blank 1, respectively. Blood extract 2 and blank 2 showed similar areas with approx. 21,000 and 24,000 AU, respectively. Thus, the impact of the blood matrix on the detection of [^13^C_5_]-5-CH_3_-H_4_folate in sample 1 and 2 was as low as 9 and 11%, respectively. The DAD chromatogram (Figure 5) shows that most of the confounders (M) are separated from analyte and internal standard. In the retention period between 12 and 16.5 min there were no detectable interfering compounds during the MS/MS Scan. Therefore, most interfering compounds obviously were removed successfully after heating, cooling, and centrifugation at 15,400 *g*. A blank chromatogram (water blank) is shown in Figure 6. Only low interferences were observed at the expected retention time of 5-CH_3_-H_4_folate (15.5–16 min). Figure 7 shows a chromatogram of the lowest calibrator concentration (0.001 nmol). Taking into account the interfering signal in Figure 6, the background contributes to less than 2% of the 5-CH_3_-H_4_folate signal (Figure 7).

#### Precision

Li and Tse (27) recommend that the bias of the precision assays should be within ±15%. Precision data for the LC-MS/MS multiple injection assay revealed a relative SD (CV) of 9.5% (114 nmol L^−1^), 7.8% (306 nmol L^−1^), and 6.9% (409 nmol L^−1^) and, thus, reproducible analysis of the samples. Compared to CVs of the multiinjection assay, we determined 3.5% for inter-day variation of a blood sample (294 nmol L^−1^) extracted in triplicate on 3 days within 3 weeks. Compared to folate analysis in DBS (18) with an inter-day variation of 3%, precision is not improved when using VAMS for determination of 5-CH_3_-H_4_folate.

#### Recoveries of SIDA

Recoveries from the spiking experiments with known amounts of 5-CH_3_-H_4_folate were 97 ± 6% (800 nmol L^−1^), 99 ± 3% (400 nmol L^−1^), and 96 ± 6% (200 nmol L^−1^) for the three spiking levels, confirming response linearity within the estimated measurement range as well as quantitative extraction and sufficient compensation of analyte losses by the internal standard.

#### Stability of 5-CH_3_-H_4_folate during Air Drying at Room Temperature and Storage at −20°C

**Figure 8:**
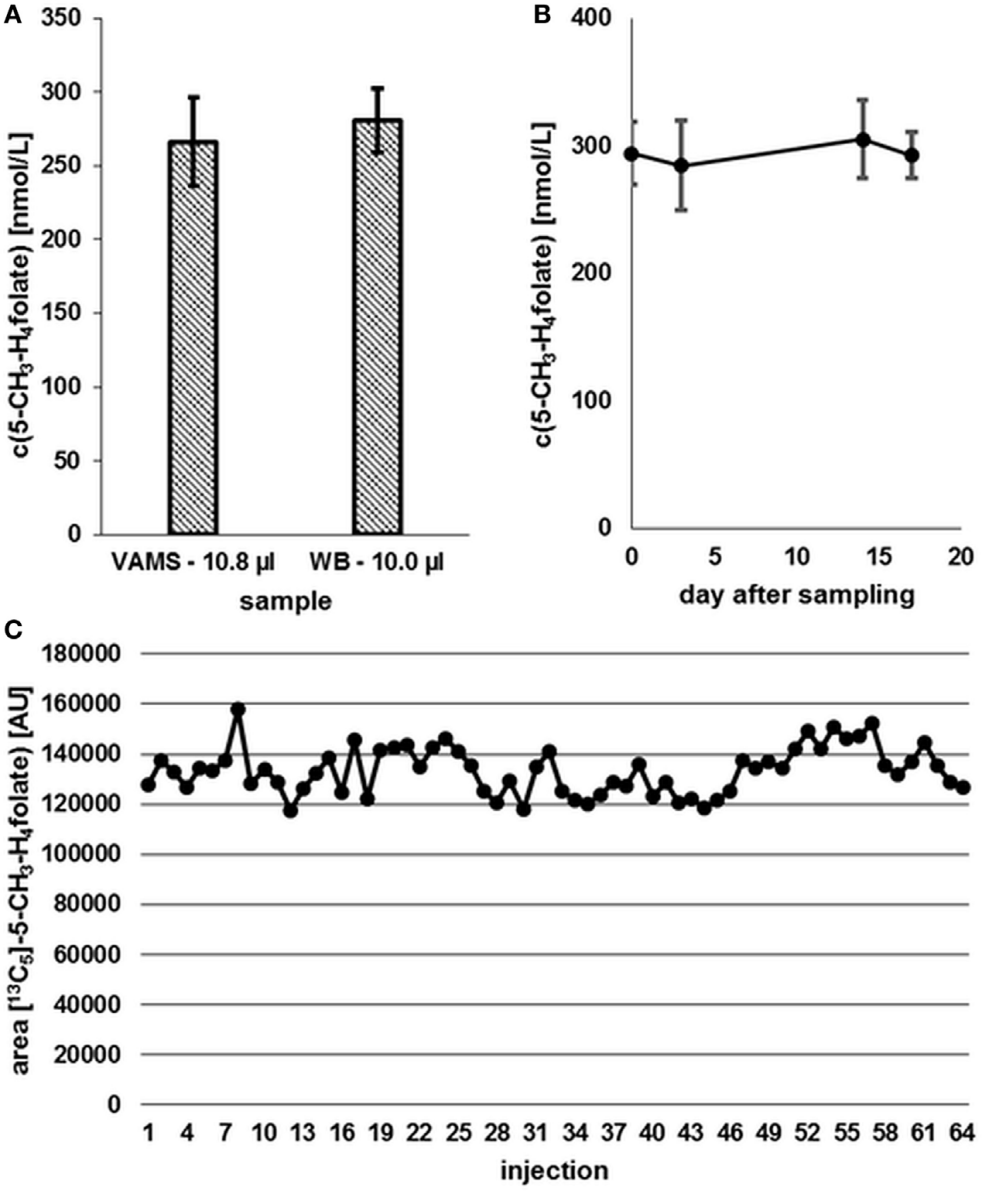
**(A)** Effect of the drying process (2.5 h) on analyte stability in volumetric absorptive microsamplers (VAMS) compared to a whole blood (WB) sample. **(B)** Stability at −20°C. **(C)** Stability in extracted samples during LC-MS/MS analysis. Finger blood from one blood donor analyzed as VAMS containing 10.8 μL of dried WB: freshly drawn WB after forefinger punction. Peak areas of [^13^C_5_]-5-CH_3_-H_4_folate obtained from multiple injections of blood sample extracts to which the same amount of [^13^C_5_]-5-CH_3_-H_4_folate has been added. **(A,B)** Mean ± SD (*n* = 3).

Compared to 10.0 μL WB samples, no significant degradation (*p* = 0.05) of 5-CH_3_-H_4_folate can be observed during the drying process of VAMS as depicted in Figure 8.

Our results (Figure 8A) indicate that the drying process has no influence on 5-CH_3_-H_4_folate stability in untreated VAMS compared to the WB sample kept at 4°C for 2.5 h. Therefore, these findings confirm stability of WB 5-CH_3_-H_4_folate in VAMS when dried at room temperature for 2.5 h. When the VAMS tip had been dried, the analyte concentration remained stable at −20°C under dry conditions and showed no significant change (*p* = 0.05) over approximately 3 weeks (Figure 8B).

#### Stability of 5-CH_3_-H_4_folate in Extracted Samples during LC-MS/MS Analysis

After 64 injections of sample extracts, we observed no loss of [^13^C_5_]-5-CH_3_-H_4_folate in a response mixture analyzed after 33 min (as first injection in the sequence) and 35.2 h (as 64th injection in the sequence). The extracts of various blood samples injected (2–63) showed no loss of [^13^C_5_]-5-CH_3_-H_4_folate as no decreasing areas were observed. Using the MES extraction buffer ensures stabilization of the analyte even at room temperature (Figure 8C).

#### Comparison of VAMS and DBS Assay

**Figure 9:**
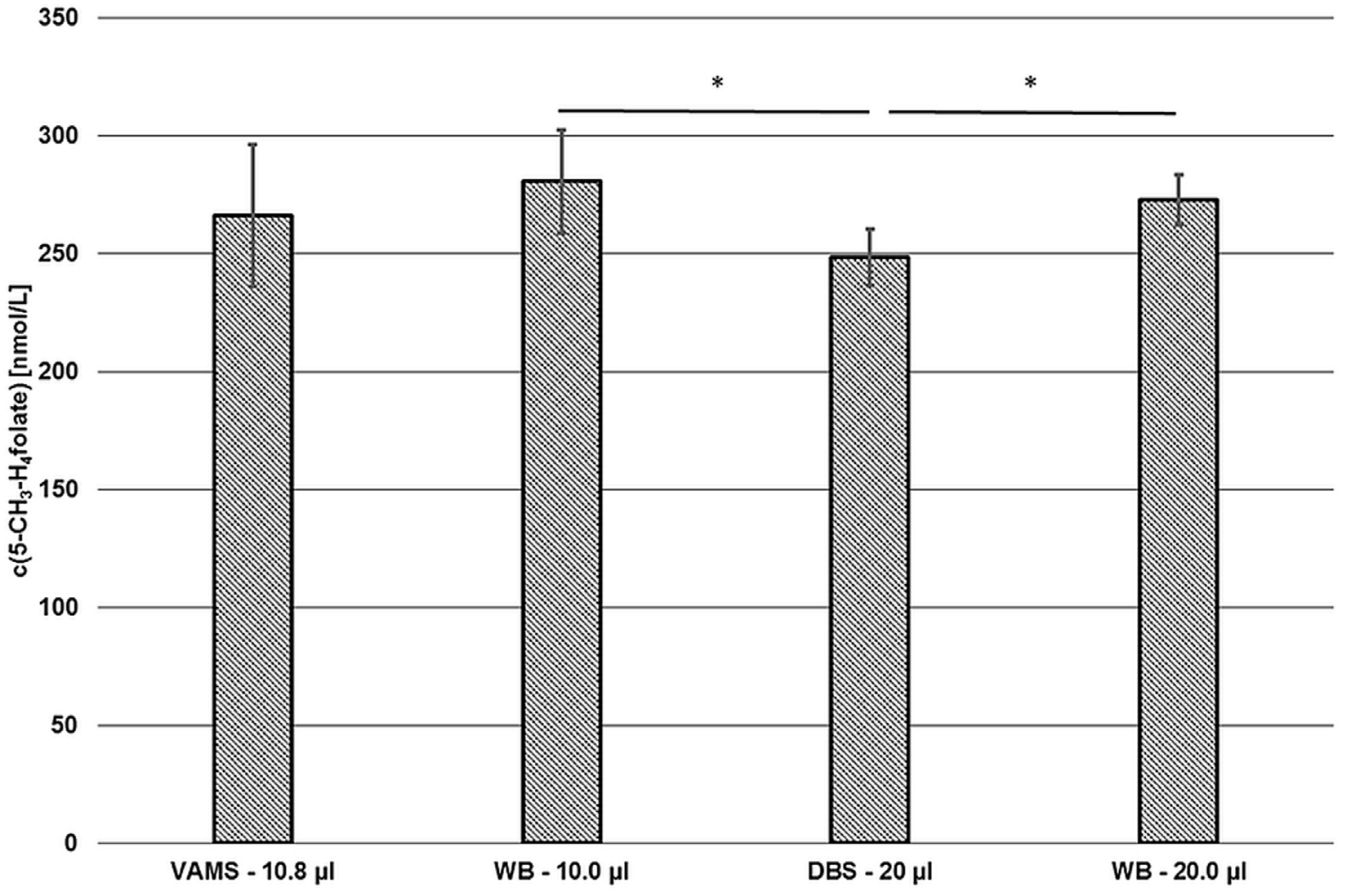
**Comparison of the volumetric absorptive microsamplers (VAMS) and the dried blood spot (DBS) assay**. VAMS—10.8 μL and whole blood (WB)—10.0 μL were extracted according to the procedure for VAMS; DBS—20.0 μL and WB—20.0 μL were extracted according to Kopp and Rychlik (18). Mean ± SD (*n* = 3). Significant difference (*p = 0.05).

Compared to our DBS assay (18), we observed no significant difference between VAMS and DBS or VAMS and venous WB (Figure 9).

About 10.0 and 20.0 μL of finger blood were pipetted in Eppendorf tubes or 15 mL centrifuge tubes and then extracted and analyzed by the extraction procedures described for VAMS (281 ± 22 nmol L^−1^) or DBS (272 ± 11 nmol L^−1^), respectively. As these results showed no significant difference (*p* = 0.05), the extraction and subsequent procedures were rated to be equivalent. Although results obtained from VAMS samplers prepared from finger blood (266 ± 30 nmol·L^−1^) were 2 and 5% lower compared to finger blood either analyzed by DBS or VAMS assay, respectively, no significant difference was observed (*p* = 0.05) as well. However, values obtained from finger blood DBS sampling (249 ± 12 nmol L^−1^) were 7, 9, and 11% lower than 5-CH_3_-H_4_folate concentrations obtained from VAMS sampling, 20.0 μL finger blood and 10.0 μL finger blood analysis, respectively. Thus, significant difference (*p* = 0.05) was observed for DBS compared to the results obtained from the finger blood analyses. Possible confounders are the procedures used for dividing the original blood sample into the aliquots used for extraction. VAMS blood is directly absorbed by the polymer, whereas DBS were produced using calibrated 20 μL-microcapillaries and WB aliquots were prepared by pipetting. Interestingly, mean 5-CH_3_-H_4_folate concentrations in VAMS in our preliminary tests were always not significantly higher than in DBS (data not shown).

### Preliminary Applications in Human Studies—Determination of WB 5-CH_3_-H_4_folate Status

**Figure 10:**
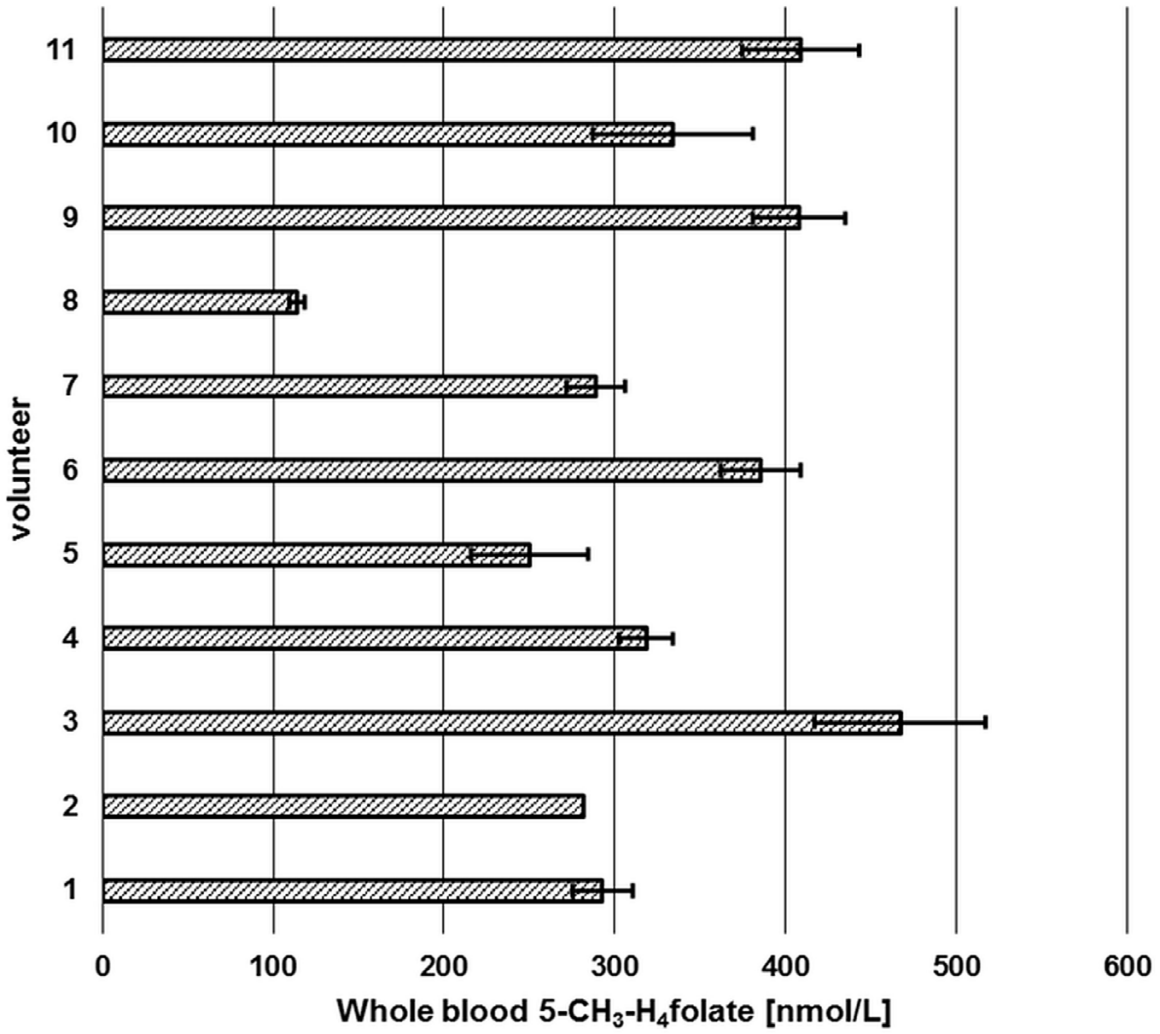
**Screening of whole blood 5-CH_3_-H_4_folate by means of volumetric absorptive microsamplers**. Mean ± SD (*n* = 3), except value for volunteer 2 (*n* = 2).

Sufficient folate uptake has to be ensured to maintain an optimal health status. According to Krawinkel et al. (31), the recommended daily intake for adults in Germany, Austria, and Switzerland is 300 μg of dietary folate equivalents (DFEs) with an additional 400 μg/day for women intending to be pregnant or during pregnancy. Obeid et al. (4) revealed an epidemic of spina bifida and anencephaly in Europe and Germany compared to the inherently lower prevalence in countries with mandatory folate fortification. About 50% of European women have a dietary folate intake of less than 200 μg DFE/day (4), which is not sufficient to prevent these diseases. Therefore, WB folate screening might be a diagnostically useful tool to assess folate status in populations to reveal further evidence for the necessity for mandatory folate fortification. We screened six female and five male volunteers for their folate status (Figure 10) in order to check our method’s suitability.

Each volunteer provided three samples. Revision of our data sets revealed that a total of two samples is necessary for precise determination of folate values. As depicted in Figure 10, triplicates from volunteer 1 to 11 (except volunteer 2, CV > 15%) were sampled correctly as all results had CVs of 5.1–13.9%. Incorrect sampling due to contact with coagulated blood on the skin or immersing the tip for more than 2 s led to 100% higher values in one of three VAMS (volunteer 2). Therefore, these results were calculated on the basis of the remaining two values, eliminating the outlier using a limit for the CV of 15% according to Li and Tse (27). The mean area of the internal standard (64 injections) was 133,531 ± 9,475 AU (CV 7.1%) reflecting the method’s stability during LC-MS/MS measurement. For clinical use and trained volunteers, two VAMS are sufficient, for untrained volunteers, we recommend the use of three VAMS. Erythrocyte folate levels of ≥340 nmol L^−1^ (≥326 nmol L^−1^ 5-CH_3_-H_4_folate) are considered adequate (31). For the limit of 326 nmol L^−1^ erythrocyte 5-CH_3_-H_4_folate, the respective critical limit for WB can be calculated as (Erythrocytes) = (DBS)/(hematocrit/100%). Normal hematocrit ranges from 40 to 54% for men and from 36 to 48% for women (32). Consequently, 326 nmol L^−1^ erythrocyte 5-CH_3_-H_4_folate corresponds to 117 and 130 nmol L^−1^ WB folate when calculated with the lowest hematocrit value for women and men, respectively, not taking into account plasma folate levels. In our pilot study, all volunteers surpassed these levels except volunteer 8 with 114 nmol L^−1^.

## Discussion

Compared to conventional folate assays, our method offers an economical alternative as no SPE and fewer reagents are required and the total time needed for extraction is reduced to less than 2 h compared to ≥8 h for usual SIDAs (15, 18). A major advantage over other WB sampling methods is the fact that the WB sample is used. Therefore, confounders like hematocrit effects on DBS filter cards, which influence the spreadability of blood and, thus, sampled volume or chromatographic effects leading to an unfavorable analyte distribution are no longer relevant (22, 33). In contrast, lower recoveries have been observed for caffeine and paraxanthine at higher hematocrit values in VAMS (34). This was attributed to the higher relative amount of erythrocytes inside the tip determining the degree of analyte desorption (34). To overcome these effects, sonication (35) was shown to improve analyte recovery at high hematocrit values. The major part of WB folate is trapped in erythrocytes. Therefore, we used 0.1% Triton X-100 as detergent to facilitate WB elution during centrifugation, which is more effective than sonication because the pores of the synthetic VAMS tip remain stable. In contrast, fast centrifugation was not suitable for our DBS folate assay (18) because of the higher sample volume (50 μL WB) and the cellulose matrix of the filter paper revealing poor pore stability. Furthermore, filter paper tends to clot and forms a compact precipitate after centrifugation and, thus, needs permanent agitation. In contrast to this, VAMS tips have a lower density and maintain their structure upon floating on the blood extract [Figure 1 (3)]. Nevertheless, the impact of hematocrit on extraction efficiency needs to be evaluated. WB samples of different hematocrit values and intact erythrocytes have to be spiked with labeled 5-CH_3_-H_4_folate because a blank matrix consisting of WB lysates treated with activated charcoal does not reflect the natural state of blood during the sampling procedure. For further validation, a certified SRM with a known amount of 5-CH_3_-H_4_folate is urgently needed to assess recovery and accuracy of WB folate assays because solely adding polyglutamate or monoglutamate does not necessarily reflect the natural state of folate in cells and plasma. Folates are most stable in their polyglutamylated form due to protein binding (36). O’Broin and Gunter (17, 37) ascribed folate stability in EDTA-DBS to the mild drying procedure at RT leading to conservation of erythrocytes with limited lysis. We assume that drying at room temperature creates a hypertonic environment and causes crenation of erythrocytes due to water efflux. In contrast to this, a pre-impregnation of DBS cards with ascorbic acid as reductant would lead to disruption of the membrane and to a release of polyglutamates being subsequently deconjugated by endogenous plasma conjugase activity and exposed to air (37). For the DBS assay, we used anticoagulated EDTA-WB spotted on filter cards (18). Surprisingly, VAMS prepared from non-stabilized finger blood showed no degradation despite substantial hemolysis can be expected during blood clotting (38, 39). After centrifugation of VAMS, we observed red and transparent solutions with no precipitates, whereas DBS from EDTA-WB showed yellow supernatants and red precipitates consisting of the cellular fraction and cellulose matrix. Therefore, we assume that stabilization with anticoagulants and the chemical composition of the absorptive matrix both influence the degree of hemolysis in these devices.

Because of a fivefold to tenfold reduction in blood specimen and a shorter conjugation step endogenous deconjugase activity was not sufficient for quantitative pteroylpolyglutamate deconjugation and, thus, exogenous deconjugase hat to be added to the sample. No significant difference (*p* = 0.05) was observed for 10.8 μL WB in VAMS when deconjugated for 1 h at 37°C and using 75 μL rat serum and 500 μL chicken pancreas suspension, compared to 20.0 μL WB or DBS when deconjugated for 4 h at 37°C with 75 μL rat serum and 1 mL chicken pancreas suspension (DBS method, Section Comparison of VAMS and DBS assay). In fact, the faster deconjugation step could be attributed to the homogeneity and uniform distribution of blood matrix after centrifugation, whereas DBS suspensions contain a larger fraction of solids, which might interfere with the deconjugation progress. Unfortunately, rat serum and chicken pancreas suspension contain significant amounts of endogenous 5-CH_3_-H_4_folate. As mentioned above (see “Preliminary Applications in Human Studies—Determination of WB 5-CH_3_-H_4_folate Status”), 117 and 130 nmol L^−1^ WB 5-CH_3_-H_4_folate are used as cutoff criterion for folate deficiency. 10 μL of WB analyzed with 75 μL rat serum and 500 μL chicken pancreas suspension contained 1 pmol endogenous enzyme 5-CH_3_-H_4_folate. In fact, almost 50% of total 5-CH_3_-H_4_folate in deconjugated blood extracts with 5-CH_3_-H_4_folate concentrations of 117 or 130 nmol L^−1^ can be attributed to the enzyme suspension. As we subtracted the background of 1 pmol 5-CH_3_-H_4_folate from each sample of our pilot study, small variations of endogenous enzyme 5-CH_3_-H_4_folate caused major variations at low WB 5-CH_3_-H_4_folate concentrations because of inhomogenous enzyme suspensions. Endogenous folate in our enzyme mixture was rather low with less than 13 nmol L^−1^ 5-CH_3_-H_4_folate in rat serum. In contrast to stabilized human plasma samples, rat serum does not contain ascorbic acid and, therefore, most of endogenous reduced folate vitamers were already oxidized or degraded in rat serum. Nevertheless, we strongly recommend the use of charcoal-stripped rat serum (40) to avoid background interferences. Hence, LOD and LOQ were determined without the use of exogenous enzyme suspension and were sufficiently low to determine 26 nmol L^−1^ 5-CH_3_-H_4_folate.

Compared to conventional assays for DBS (18) and erythrocytes (15), the SPE step was omitted. A major drawback of the SPE step is the loss of 43% 5-CH_3_-H_4_folate during purification (18). Using only 10.8 μL WB for extraction and a long column for chromatography, most of the matrix could be separated completely from 5-CH_3_-H_4_folate and its internal standard during LC-MS/MS measurement. Therefore, LOD and LOQ of DBS (18) and erythrocytes (15) are comparable to those obtained from VAMS despite a fivefold to tenfold reduced sample volume. In clinical routine analytics, modern UPLC-MS/MS methods could substantially minimize the duration of each LC-MS/MS cycle, especially when more than one VAMS has to be analyzed.

Costs for DBS assays with 100 filter cards (500 spots) and 500 SPE cartridges amount to approximately 930$ compared to 580$ (−38%) for 500 VAMS (125 clamshells). Costs for chemicals are 50% lower if the VAMS assay is used. Moreover, this method is suitable for automatization and might be miniaturized further by using fast UPLC-MS/MS techniques for separation. Furthermore, volumetric absorptive microsampling constitutes a direct sampling technique, whereas DBS self-sampling needs more material (lancets, EDTA-coated tubes, microcapillaries), which makes it even more expensive.

This paper presents a first approach for assaying 5-CH_3_-H_4_folate in 10.8 μL dried WB. WB collection should be carried out by trained individuals and no fasting is required. Whether automated or not, the method described herein can be used as rapid testing for folate status in humans and women of childbearing age.

## Ethics Statement

Blood sampling of the volunteers was performed after written informed consent of the volunteers with permission of the Ethics Commission of the TUM School of Medicine of the Technical University of Munich (project 69/16 S).

## Author Contributions

MK and MR were responsible for the study design. Furthermore, these authors contributed to the correction and improvement of the manuscript. MR mentored MK and was responsible for funding, data interpretation, and the manuscript. MK was responsible for the development and validation of all methods and statistical analyses for the data utilized for this manuscript, and wrote and formatted the manuscript. All authors mentioned read the manuscript and implemented their improvements.

## Conflict of Interest Statement

The authors declare that the research was conducted in the absence of any commercial or financial relationships that could be construed as a potential conflict of interest.
